# Evaluation of the Making Employment Needs (MEN) Count Intervention to Reduce HIV/STI Risk for Black Heterosexual Men in Washington DC

**DOI:** 10.1177/1557988319869493

**Published:** 2019-08-21

**Authors:** Anita Raj, Nicole E. Johns, Florin Vaida, Lianne Urada, Jenne Massie, Jennifer B. Yore, Lisa Bowleg

**Affiliations:** 1 Center on Gender Equity and Health, Department of Medicine, University of California San Diego, La Jolla, CA, USA; 2 Department of Education Studies, Division of Social Sciences, University of California San Diego, La Jolla, CA, USA; 3 School of Social Work, San Diego State University, USA; 4 George Washington University, Washington, DC, USA

**Keywords:** HIV/AIDS, sexually transmitted diseases/infections, social determinants of health, psychosocial and cultural issues, masculinity, gender issues and sexual orientation, employment issues, occupational health

## Abstract

The primary aim of this study was to evaluate the impact of MEN Count, a race- and gender-tailored three-session counseling intervention, on HIV/STI incidence as well as housing and employment. A two-armed quasi-experimental design was used to compare MEN Count to an attention comparison condition focused on stress reduction, from March 2014 to April 2017. Participants (*N* = 454) were Black heterosexual men in Washington DC, largely recruited from an STI clinic. Multivariate difference-in-difference regressions assessed whether the intervention was associated with significant changes in the outcomes set, which included nonviral STI incidence, sexual risk categorization, housing, and employment. Significant improvements over time were observed across both treatment arms for all outcomes (*p* < .05). Reductions in unemployment were significantly greater for intervention than for control participants (AOR unemployment = 0.48, 95% CI [0.23, 0.99]). Improvements in other outcomes did not differ significantly by treatment group. In dose analyses, participants receiving all intervention sessions were significantly less likely than control participants to have experienced homelessness in the 90 days prior (AOR= 0.31, 95% CI [0.10, 0.96]) and to be unemployed (AOR = 0.37, 95% CI [0.14, 0.96]). The MEN Count intervention offers a promising approach to address structural risk factors for STI, but not STI itself, among this largely STI clinic–based sample.

The human immunodeficiency virus (HIV) and sexually transmitted infection (STI) epidemics in the United States disproportionately affect Black communities (CDC, 2016, 2017a, 2017b, November, 2017), contributing to inequities in infertility (Bitler & Schmidt, 2006) and early mortality (Siddiqi, Hu, & Hall, 2015). Rates of HIV/STI vary geographically and are among the highest in the nation in Washington, District of Columbia (DC; CDC, 2016, 2017a), a metropolitan area where almost half the population is Black (“QuickFacts - United States: U.S. Census 2016 Projections,” 2010 - 2017). Washington DC has a 2% HIV prevalence rate, indicative of a generalized HIV epidemic, and among Black men in Washington DC, this rate is 4.4% (HAHSTA, 2017). STIs among men are also two to four times higher in Washington DC than seen nationally (CDC, 2017a). Among those living with HIV in Washington DC, 1 in 10 Black men and the majority of Black women acquired HIV via heterosexual sex (HAHSTA, 2017).

HIV “test and treat” interventions emphasize identification of those infected with HIV and support of their medical adherence and viral suppression (Mayer, 2011). While these are important to address the HIV epidemic, “test and treat” models alone may be inadequate to prevent transmission of all STIs. For populations at increased risk for STI and HIV, such as Black men in Washington, DC, primary prevention efforts remain beneficial, particularly when they address the structural and contextual risk associated with marginalization at the intersections of race, gender, and economic deprivation (Bowleg & Raj, 2012; Raj & Bowleg, 2012). This study involves the implementation and evaluation of a race- and gender-tailored HIV/STI prevention program for Black heterosexual men in Washington DC: Making Employment Needs (MEN) Count. MEN Count was designed to promote safer sex, reduce STI, and help support stabilized housing and employment, as these are recognized as key structural risks associated with HIV/STI among Black heterosexual men (Bowleg & Raj, 2012; Raj & Bowleg, 2012).

## Methods

### Study Design

A two-armed evaluation trial was conducted among Black heterosexual men reporting structural and sexual risks (see risk criteria in the following text) for HIV/STI to evaluate the effects of the MEN Count intervention on STI incidence within this population, and secondarily on sexual risk, housing, and employment. Participants (*N* = 454) were recruited from an STI clinic and via participant referral and community outreach in Washington DC from August 2014 to April 2017.

The study compared MEN Count to a similarly structured control condition. MEN Count involved three gender-tailored counseling sessions delivered by a male peer case manager and focused on sexual risk and relationships, as well as housing and employment stability. Control participants received a case manager–delivered program of similar structure and length, focused on stress management. The study was quasi-experimental, with participants recruited equivalently across arms and assigned to the intervention or control condition based on case manager availability at the time of recruitment. There was no set pattern for case manager availability.

### Sample and Recruitment

Participants were recruited via community and street outreach, flyers and Craigslist, participant referrals, and on-site recruitment at a large publicly funded STI clinic. Of the 1,042 participants screened to assess eligibility, 595 were identified as eligible. Eligible participants were self-identified Black men aged 18 years and older, reporting heterosexual risk for HIV/STI (defined as unprotected sex with a woman AND two or more female sex partners in the past year) and who were either currently unemployed or had experienced homelessness in the previous 6 months. Of these 595 eligible men, 455 participants (76.5% participation rate) provided written informed consent and enrolled in the study; 1 participant was withdrawn from the study due to use of false information, resulting in a final sample of *N* = 454.

**Figure 1.**
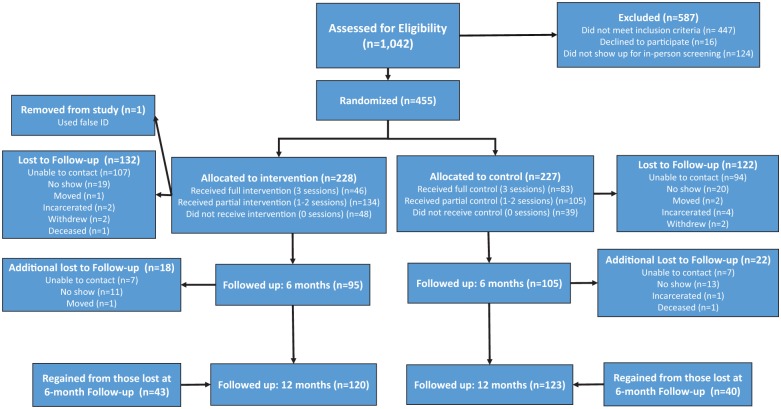
Consort flow chart.

Participants (*n* = 227 intervention, *n* = 227 control) were surveyed and tested for HIV and STIs at baseline and 6- and 12-month follow-up. Follow-up data were obtained from 44.1% (*n* = 200) and 53.5% (*n* = 243) of participants at the 6- and 12-month follow-ups, respectively. Loss to follow-up was primarily due to inability to locate the participant. Seven individuals were lost due to incarceration and two participants died during the course of the study. See Figure 1 for more details.

### Study Procedure

Trained research staff conducted all screening for eligibility and study recruitment. Once consented and enrolled in the study, participants were assigned to a treatment group, escorted to a private room for the baseline assessment, and, subsequent to baseline data collection, linked to the case manager to receive the intervention or comparison group case management sessions. Baseline data collection involved trained research staff using mobile tablets to collect detailed survey data on participants’ demographics, risk behaviors, HIV/STI risk profile, and HIV/STI knowledge, attitudes, and risk perceptions. Clinic and study staff also conducted standard of care HIV/STI counseling and testing as part of the baseline assessment; this included support for follow-up and linkage to care or treatment if a participant received a positive HIV/STI test result. These same procedures were used for survey and HIV/STI testing at 6- and 12- month follow-ups for all study participants. Participants received $30 cash at baseline, $40 at 6-month follow up, and $50 at 12-month follow up for their study participation.

### Data Management

Staff uploaded survey data directly from tablets to a secure server system, and HIV/STI test results obtained from the STI/HIV testing site were linked to survey data using unique identifiers to maintain participant confidentiality. These identifiers were also used to link baseline and follow-up data.

### MEN Count Intervention

As discussed elsewhere (Raj et al., 2014), the intervention is based on the Social Cognitive Theory (Bandura, 1990) and the Theory of Gender and Power (Connell, 1987). Collectively, these frameworks consider social–cognitive, structural, and gendered risks for HIV. The intervention was designed to be gender transformative, in that traditional gender norms underlying risk behavior were questioned and reconsidered as part of the counseling sessions (WHO, 2011). More specifically, the intervention addressed conventional restrictive masculinity ideologies, such as those related to hypersexuality, invincibility, and dominance, that reinforce men’s harmful and risky behaviors, as well as those related to their HIV/STI risk (e.g., sex trade involvement, substance use) and perpetration of violence in relationships. It was designed to also address structural factors affecting health, specifically homelessness and unemployment. Case managers worked with clients to consider their risk for HIV/STIs, build action plans to change behavior and achieve goals, and update and validate achievements. They also discussed housing and employment situations and provided social support and resources for these as needed.

The intervention entailed three 1-hour sessions of one-on-one case management, involving risk reduction counseling integrated with employment and housing case management, delivered over a timeframe of 60–90 days. Brief check-in sessions were included as needed or requested by participants. Peer case managers conducted sessions in a private location, and Sessions 2 and 3 could be conducted by phone if the client could not otherwise return. Study authors previously conducted a one-armed feasibility trial evaluating MEN Count using baseline and 6-month follow-up data and reported a significant reduction in unsafe sex and homelessness and significant improvement in employment (Raj et al., 2014). This data showed the promise of the Men Count model for the current study.

#### Peer case manager training and quality assurance methods

MEN Count peer case managers received week-long training on HIV/STI prevention counseling, health consequences of harmful masculinity ideologies, healthy relationships and intimate partner violence prevention, and case management. A doctoral-level social worker supervised monthly meetings to review cases. All sessions were audio-recorded and 10% of these were reviewed on an ongoing basis for quality control. Additionally, participants completed a brief survey at follow-up about their response to the intervention program; responses were largely favorable (see Web Table S4). Based on this approach to monitoring and quality assurance checks (Bellg et al., 2004), case managers were supported via monthly supervisor meetings.

### Control Condition

To address the potential for Hawthorne effects, that is, the effects of attention from the intervention, or in this case the case manager (McCarney et al., 2007), the control condition maintained a case management program similar in length to MEN Count but with a focus on stress management.

### Measures

#### Outcome variables

The primary outcome was diagnosis of a nonviral STI from STI clinic test results. The STI clinic tested for the following nonviral STIs: chlamydia, gonorrhea, syphilis, and atypical urethritis (AU). AU testing was not available to a subsample of participants who received testing for STIs outside the STI clinic at baseline (*n* = 64). The STI clinic provided treatment for all nonviral STIs, so all diagnoses were assumed to be incident cases. The STI clinic also tested for HIV and herpes simplex, although these were excluded due to inability to consider reinfections and incidence at follow-up. As nonviral STI incidence was selected as the primary outcome, STI incidence inclusive of viral STIs was also assessed as a robustness check. Inclusion of viral STI diagnosis changed the STI outcome for one individual at one time point, and therefore exclusion of viral STI diagnosis was not considered meaningful.

Secondary outcomes included sexual risk for HIV/STI, unemployment, and homelessness.

Sexual risk was constructed from survey items on past 90-day number of female sex partners (vaginal or anal sex), consistency of condom use during vaginal or anal sex with female partner(s), and participation in transactional sex.

Homelessness was characterized as at least one night of homelessness in the past 90 days. Per the U.S. Department of Health and Human Services definition (NHCHC, n.d.), participants were categorized as having experienced homelessness if they answered “homeless on the streets” or “homeless in a shelter” in response to the question “What best describes your living situation in the past 90 days?” or if they had a non-zero response to either of two questions asking how many of the previous 90 days the participant was homeless on the streets or in a shelter.

Employment was assessed via a single item asking the participant’s current employment status. Participants could indicate that they were not employed, were not legally employed but had a job with income, or were legally employed full-time or part-time. Participants reporting that they were illegally employed were categorized as unemployed.

#### Independent variables

The primary independent variable was treatment group: MEN Count or control.

Dose analyses were conducted to determine intervention effects based on number of counseling sessions received. Of 227 intervention-assigned participants, 21% (*n* = 48) attended no MEN Count counseling sessions; 47% (*n* = 106) attended one; 12% (*n* = 27) attended two; and 20% (*n* = 46) attended all three sessions. Of 227 control-assigned participants, 17% (*n* = 39) attended no stress reduction counseling sessions; 22% (*n* = 50) attended one; 24% (*n* = 55) attended two; and 37% (*n* = 83) attended all three sessions. Session attendance was significantly higher for control relative to intervention participants.

#### Covariates

Time and Time by Treatment: Time point of survey was classified as 0, 1, and 2 for baseline, 6-month, and 12-month follow-up, respectively. A treatment–time interaction was generated as 1 for those in the intervention group at follow-up and 0 otherwise.

Demographics: Demographics at baseline included age, highest level of education, and history of incarceration (categorized as never, not in the past 90 days, in the past 90 days).

Recruitment location/mode: Craigslist, STI clinic, friend referral, other.

### Data Analysis

Difference-in-difference models using mixed-effect logistic regression were constructed to assess the impact of the intervention on nonviral STI incidence and homelessness. A random intercept for individual was included to account for repeated measurements over time. Difference-in-difference models using multinomial logistic regression were constructed for the three-level employment variable and four-level sexual risk variable, clustering by individual to account for repeated measurements over time. Demographic covariates were included if they were associated with the treatment group at baseline in bivariate chi-squared tests at *p* < .20; education, employment, and recruitment source were included as a result. Baseline employment was not included in the respective outcome model. For each outcome, additional potential covariates were included if they were associated with the given outcome in bivariate chi-squared tests at *p* < .05. All models included time, treatment, and the time–treatment interaction. These intent-to-treat analyses were repeated using a dose–time interaction to assess possible dose response. For dose analyses, control participants were classified as receiving no sessions. The effect of attendance for *any* type of counseling session was further tested via inclusion of number of sessions attended (either control or intervention) as a covariate in adjusted models.

All analyses were conducted using STATA 15.1. Significance was set at *p* < .05 for all chi-square tests and adjusted odds ratios (AORs); 95% confidence intervals (CIs) are reported throughout.

All procedures were reviewed and approved by the Institutional Review Boards of the University of California San Diego, the George Washington University, as well as the DC Department of Health Institutional Review Board for the Public Health. This study was registered with clinicaltrials.gov on September 26, 2012 (Clinical Trials number NCT101694121).

## Results

### Characteristics of the Sample at Baseline

In total, 454 men were included in the current analyses (Table 1, Web Table S1). The average age of participants was 31 years (*SD* 10.1, range 18–65). One in six (*n* = 74, 16.3%) had not completed high school or obtained a General Education Development (GED) diploma. Three quarters (*n* = 318, 71.6%) had been incarcerated in their lifetime, 15% (*n* = 68) in the past 90 days. Half (*n* = 220, 48.6%) of participants had been homeless in the past 90 days, and two thirds (*n* = 307, 67.8%) were unemployed. A total of 115 participants (27% of those with STI testing data at baseline) tested positive for a nonviral or viral STI, including seven HIV-positive individuals. One quarter of participants (*n* = 106, 24.5%) tested positive for a nonviral STI, which serves as the basis of the outcome for evaluation. At baseline and in reference to the previous 90 days, 11% of participants with nonmissing data (*n* = 37) reported consistent condom use, 77% (*n* = 349) reported having two or more female partners, 23% (*n* = 105) had engaged in transactional sex, and 3% (*n* = 13) reported having had sex with a man (results not presented).

**Table 1.** Characteristics at Baseline of MEN Count Participants, Overall and by Treatment Condition (*N* = 454).^a^

Characteristic	Total sample *n* (%)	Control *n* (%)	Intervention *n* (%)	Chi-squared test *p* value
*N*	454 (100)	277 (100)	277 (100)	
*Demographics*				
Age				.273
18–24	134 (29.5)	65 (28.6)	69 (30.4)	
25–29	127 (28.0)	70 (30.8)	57 (25.1)	
30–39	120 (26.4)	62 (27.3)	58 (25.6)	
40–65	73 (16.1)	30 (13.2)	43 (18.9)	
Recruitment source				.032
At clinic	243 (53.5)	124 (54.6)	119 (52.4)	
Friend	111 (24.5)	53 (23.3)	58 (25.6)	
Craigslist	64 (14.1)	39 (17.2)	25 (11.0)	
Flyer, CBO, other	36 (7.9)	11 (4.8)	25 (11.0)	
Education				<.001
Less than HS diploma/GED	74 (16.3)	45 (19.8)	29 (12.8)	
GED	78 (17.2)	49 (21.6)	29 (12.8)	
HS diploma	144 (31.7)	53 (23.4)	91 (40.1)	
Some college or more	158 (34.8)	80 (35.2)	78 (34.4)	
Incarcerated ever				.763
No	126 (28.4)	61 (27.7)	65 (29.0)	
Yes	318 (71.6)	159 (72.3)	159 (71.0)	
Incarcerated in past 90 days				.430
No	386 (85.0)	196 (86.3)	190 (83.7)	
Yes	68 (15.0)	31 (13.7)	37 (16.3)	
*Outcomes at baseline*				
Nonviral STI				.820
No	326 (75.5)	161 (75.9)	165 (75.0)	
Yes	106 (24.5)	51 (24.1)	55 (25.0)	
Sexual risk				.110
Very low	29 (7.9)	10 (5.6)	19 (10.2)	
Low	84 (22.9)	36 (20.0)	48 (25.7)	
Medium	191 (52.0)	104 (57.8)	87 (46.5)	
High	63 (17.2)	30 (16.7)	33 (17.6)	
Homeless in past 90 days				.279
No	233 (51.4)	122 (54.0)	111 (48.9)	
Yes	220 (48.6)	104 (46.0)	116 (51.1)	
Current employment				.081
Unemployed	307 (67.8)	149 (65.9)	158 (69.6)	
Employed part-time	93 (20.5)	43 (19.0)	50 (22.0)	
Employed full-time	53 (11.7)	34 (15.0)	19 (8.4)	

*Note*. CBO = community-based organization; GED = General Education Development; HS = high school; MEN = Making Employment Needs; STI = sexually transmitted infection.

a Missing values not reported.

### Differences Between Treatment Groups at Baseline

There were no significant differences between treatment groups for nonviral STI or level of sexual risk at baseline (Table 1). There were significant differences between treatment groups in demographics at baseline, specifically for education (control more likely than intervention to have less than a high school diploma (19.8% vs. 12.8%) or have a GED (21.6% vs. 12.8%, *p* < .001), employment (control more likely than intervention to be employed full-time [15.0% vs. 8.4%] *p* = .08) and recruitment source (control more likely than intervention to be recruited via Craigslist [17.2% vs. 11.0%]) and less likely via flyer or other recruitment mechanism (4.8% vs. 11.0%, *p* = .03) at *p* < .20; these factors were thus included in the adjusted outcome analyses. Age was also included as a covariate, despite no significant difference by treatment group at baseline.

### Differences by Study Retention

Study retention rates were very low; of 454 participants, 44.1% (*n* = 200) and 46.5% (*n* = 211) completed the 6- and 12-month follow-ups, respectively (see Figure 1). Retention did not differ significantly between treatment and control arms at 6-month (41.9% vs. 46.3%, *p* = .34) or 12-month follow-up (52.9% vs. 54.2%, *p* = .74). Reasons for loss to follow-up were predominantly inability to contact and no-shows for scheduled surveys. Additionally, four participants withdrew from the study and two died during the study period.

In analyses assessing differences in retention, those lost to follow-up were more likely than those retained to have reported recent incarceration at baseline (20.5% vs. 11.7%, *p* = .01) and to have been recruited in clinic, via flyer, or community-based organization outreach rather than via participant referral or Craigslist (70.8% vs. 55.9%, *p* = .01; see Web Table S2). Past 90-day incarceration was thus added as a covariate in adjusted models; recruitment mechanism was already included due to association with treatment conditions. No other tested variables were associated with study retention.

### Difference-in-Difference Analyses to Evaluate Outcome Effects

All outcomes saw statistically significant (*p* < .05) improvement over time for both intervention and control groups at 12 months. However, there was no significant time-by-treatment effect on nonviral STI incidence, sexual risk categorization, or homelessness (Tables 2 and 3). A significant time-by-treatment interaction effect was seen for unemployment relative to full-time employment (*p* = .046; Table 3). Those in the treatment group had half the odds (AOR = 0.48, 95% CI [0.23, 0.99]) of being unemployed relative to being employed full-time at follow-up than those in the control condition, accounting for baseline rates of employment and the overall change in employment over time for the study population. Part-time employment did not have a significant time-by-treatment effect. Further examination of the time-by-treatment effect on unemployment revealed that unemployment was significantly lower in the intervention relative to control group at 6-month follow-up (AOR = 0.32, 95% CI [0.14, 0.76], *p* = .01). However, treatment groups did not significantly differ at 12-month follow-up when the follow-up time points were considered independently (AOR = 0.61, 95% CI [0.28, 1.35], *p* = .28; results not presented).

**Table 2.** Mixed Effects and Multinomial Logistic Regression Models Assessing the Effect of the MEN Count Intervention on Nonviral STI Incidence (Reference Is No STI Diagnosis, *N* = 448) and Sexual Risk Behavior Categorization (Reference Is Very Low Risk, *N* = 367).

	STI^a^		Sexual risk behaviors^b^					
			High		Medium		Low	
	AOR	95% CI	AOR	95% CI	AOR	95% CI	AOR	95% CI
Treatment–time interaction	0.78	[0.32, 1.90]	0.92	[0.27, 3.06]	1.57	[0.58, 4.24]	0.92	[0.32, 2.59]
Treatment								
Control	*Ref*	*Ref*	*Ref*	*Ref*	*Ref*	*Ref*	*Ref*	*Ref*
Intervention	1.19	[0.65, 2.19]	0.60	[0.23, 1.54]	0.48	[0.20, 1.11]	0.73	[0.30, 1.80]
Time								
Baseline	*Ref*	*Ref*	*Ref*	*Ref*	*Ref*	*Ref*	*Ref*	*Ref*
6-month follow-up	0.36**	[0.17, 0.77]	0.19**	[0.07, 0.53]	0.18***	[0.08, 0.44]	0.62	[0.25, 1.53]
12-month follow-up	0.48*	[0.24, 0.96]	0.15***	[0.06, 0.38]	0.15***	[0.07, 0.35]	0.48	[0.20, 1.14]
Age								
18–24	*Ref*	*Ref*	*Ref*	*Ref*	*Ref*	*Ref*	*Ref*	*Ref*
25–29	1.68	[0.90, 3.13]	1.33	[0.52, 3.40]	1.63	[0.79, 3.39]	1.45	[0.72, 2.93]
30–39	0.65	[0.33, 1.31]	1.26	[0.48, 3.28]	1.19	[0.57, 2.48]	1.16	[0.54, 2.53]
40–65	0.43	[0.16, 1.14]	0.89	[0.31, 2.56]	0.59	[0.25, 1.40]	0.92	[0.42, 2.00]
Recruitment source								
At clinic	*Ref*	*Ref*	*Ref*	*Ref*	*Ref*	*Ref*	*Ref*	*Ref*
Friend	0.14***	[0.06, 0.30]	1.84	[0.78, 4.33]	0.99	[0.50, 1.97]	1.41	[0.73, 2.74]
Craigslist	0.33**	[0.15, 0.75]	0.62	[0.22,1.74]	0.94	[0.42, 2.07]	1.11	[0.54, 2.29]
Flyer, CBO, other	0.23*	[0.06, 0.82]	1.87	[0.50, 7.05]	0.89	[0.29, 2.69]	1.40	[0.52, 3.78]
Education								
Less than HS diploma/GED	0.87	[0.39, 1.93]	1.60	[0.55, 4.64]	1.17	[0.51, 2.69]	1.28	[0.54, 3.04]
GED	1.08	[0.52, 2.27]	3.96*	[1.27, 12.33]	1.86	[0.74, 4.70]	2.09	[0.87, 5.00]
HS diploma	*Ref*	*Ref*	*Ref*	*Ref*	*Ref*	*Ref*	*Ref*	*Ref*
Some college or more	0.69	[0.36, 1.32]	2.04	[0.85, 4.90]	1.46	[0.76, 2.83]	1.18	[0.64, 2.20]
Employed at baseline								
Unemployed	*Ref*	*Ref*	*Ref*	*Ref*	*Ref*	*Ref*	*Ref*	*Ref*
Employed part-time	0.42*	[0.21, 0.85]	1.32	[0.54, 3.23]	1.15	[0.59, 2.23]	1.59	[0.87, 2.92]
Employed full-time	1.15	[0.54, 2.46]	0.69	[0.23, 2.05]	0.74	[0.31, 1.78]	0.65	[0.25, 1.65]
Incarcerated in past 90 days at baseline								
No	*Ref*	*Ref*	*Ref*	*Ref*	*Ref*	*Ref*	*Ref*	*Ref*
Yes	1.02	[0.50, 2.09]	2.07	[0.79, 5.43]	2.75*	[1.02, 7.40]	1.85	[0.71, 4.84]

*Note*. AOR = adjusted odds ratio; CBO = community-based organization; GED = General Education Development; HS = high school; MEN = Making Employment Needs; STI = sexually transmitted infection.

a Model includes random effect on individual. ^b^Model clustered on individual.

* *p* < .05. **p < .01. ***p < .001.

**Table 3.** Multinomial and Mixed-Effects Logistic Regression Models Assessing the Effect of the MEN Count Intervention on Employment (Reference Is Full-Time Employment, *N* = 453) and Homelessness (Reference Is No Homelessness in the Prior 90 Days, *N* = 452).

	Employment^a^				Homelessness^b^	
	Unemployed		Employed Part-Time			
	AOR	95% CI	AOR	95% CI	AOR	95% CI
Treatment–time interaction	0.48*	[0.23, 0.99]	0.73	[0.30, 1.74]	0.76	[0.33, 1.71]
Treatment						
Control	*Ref*	*Ref*	*Ref*	*Ref*	*Ref*	*Ref*
Intervention	1.73	[0.91, 3.27]	1.92	[0.94, 3.89]	1.25	[0.65, 2.41]
Time						
Baseline	*Ref*	*Ref*	*Ref*	*Ref*	*Ref*	*Ref*
6-month follow-up	0.62*	[0.38, 1.00]	0.63	[0.33, 1.21]	0.26***	[0.13, 0.51]
12-month follow-up	0.43***	[0.26, 0.71]	0.52*	[0.27, 0.99]	0.24***	[0.12, 0.47]
Age						
18–24	*Ref*	*Ref*	*Ref*	*Ref*	*Ref*	*Ref*
25–29	0.70	[0.40, 1.25]	0.73	[0.40, 1.33]	1.89	[0.89, 4.01]
30–39	1.20	[0.63, 2.28]	0.67	[0.34, 1.32]	2.79**	[1.28, 6.09]
40–65	1.34	[0.57, 3.15]	0.76	[0.32, 1.80]	5.28***	[2.04,13.66]
Recruitment source						
At clinic	*Ref*	*Ref*	*Ref*	*Ref*	*Ref*	*Ref*
Friend	1.70	[0.85, 3.38]	1.24	[0.56, 2.72]	5.09***	[2.42, 10.70]
Craigslist	0.65	[0.34, 1.23]	1.27	[0.66, 2.42]	1.71	[0.74, 3.98]
Flyer, CBO, other	0.60	[0.22, 1.61]	1.41	[0.55, 3.63]	6.69**	[2.07, 21.55]
Education						
Less than HS/GED	1.15	[0.52, 2.57]	0.76	[0.31, 1.88]	1.05	[0.44, 2.51]
GED	0.58	[0.29, 1.18]	0.64	[0.28, 1.45]	2.65*	[1.14, 6.14]
HS diploma	*Ref*	*Ref*	*Ref*	*Ref*	*Ref*	*Ref*
Some college or more	0.59	[0.34, 1.05]	0.93	[0.53, 1.65]	0.66	[0.32, 1.34]
Incarcerated in past 90 days at baseline						
No	*Ref*	*Ref*	*Ref*	*Ref*	*Ref*	*Ref*
Yes	2.00**	[1.20, 3.30]	1.20	[0.70, 2.06]	1.50	[0.68, 3.29]
Homeless in past 90 days at baseline						
No	*Ref*	*Ref*	*Ref*	*Ref*		
Yes	1.65	[0.73, 3.73]	1.19	[0.52, 2.76]		
Employed at baseline						
Unemployed					*Ref*	*Ref*
Employed part-Time					0.30**	[0.14, 0.63]
Employed full Time					0.42	[0.17, 1.06]

*Note*. AOR = adjusted odds ratio; CBO = community-based organization; GED = General Education Development; HS = high school; MEN = Making Employment Needs; STI = sexually transmitted infection.

a Model clustered on individual. ^b^Model includes random effect on individual.

* *p* < .05. **p < .01. ***p < .001.

### Dose Analyses

There were no significant dose effects on STI incidence or sexual risk categorization. However, men receiving the full MEN Count intervention (three sessions) were significantly less likely than those receiving no session to have experienced homelessness in the prior 90 days at follow-up (AOR = 0.31, 95% CI [0.10, 0.96], *p* = .04) and significantly less likely to be unemployed relative to full-time employed at follow-up (AOR = 0.37, 95% CI [0.14, 0.96], *p* = .04; see Web Table S3).

### Exploratory Analysis of Financial Impact of Employment Effects

Given the improvement in full-time employment among participants of the MEN Count program, a post hoc exploratory analysis was conducted to compute projected lifetime financial benefits of full-time employment for study participants, taking into account observed average income by employment status, current age and likely retirement age, and a risk-free rate of discounting based on 3-month treasury bill rates at the time of study. Monthly average income for full-time employment and unemployment were derived from participants’ reports of income and determined to be $1,787.87 for those employed full-time and $270.66 for those unemployed or illegally employed. The probability of maintaining full-time employment was derived using participant reports of employment at baseline and follow-up and determined to be 0.6. Average age of study participants at follow-up was 31 years and retirement age was assumed to be 65 years. The risk-free rate for discounting was assumed to be 2.09%, based on the 3-month treasury bill rate as of August 23, 2018 (US-Treasury, 2018). The present discounted value of lifetime earnings was thus calculated to be $78,484.83 for those who were unemployed and $353,377.35 who were employed full-time at follow-up. Therefore, effects of MEN Count can support full-time employment at a level that can potentially support participants’ net lifetime benefit of $274,892.52.

## Discussion

Findings from this study indicate that MEN Count, a gender-tailored case management–delivered HIV/STI prevention intervention, had no significant effect on incident STI or sexual risk among Black heterosexual men. This is disappointing in light of the sharp increases in U.S. STI rates seen each year in the past 4 years (CDC, August 2018). Study findings are inconsistent with those seen in a recent meta-analysis of effective HIV/STI interventions for Black heterosexual men, which indicated that gender- and culture-tailored interventions linked to medical services, delivered by male facilitators, and supporting men with an incarceration history have a significant impact on HIV/STI risk reduction (Henny et al., 2012). Findings are consistent with prior research indicating that few prevention interventions in STI clinics demonstrate significant impact on STIs (Long et al., 2016). Possibly, this is because the STI clinic counseling and testing environment itself has an effect, in the sense that STI clinic attendees are at increased awareness of STI risk at time of attendance; across treatment conditions there were significant reductions in STI incidence and level of sexual risk.

MEN Count did demonstrate significant improvements in employment and, for those with higher session attendance and housing stability, in line with the findings from the pilot MEN Count study (Raj et al., 2014). The value of this approach in supporting these structural factors affecting health—namely, employment and housing—cannot be understated, given the disproportionate burden Black men face on both issues (“QuickFacts - United States: U.S. Census 2016 Projections,” 2010 - 2017). Exploratory economic calculations conducted to determine discounted value of lifetime earnings for MEN Count participants suggest that these effects on employment can potentially support a participant’s net lifetime benefit of $274,892.52, even accounting for underemployment and unstable employment of the study population. Such findings support the paradigm of addressing structural needs to improve health and should be expanded to consider how health infrastructures might be better used to reach and address these determinants and support vulnerable populations more holistically.

While these findings show promise, they must be considered in light of major study limitations, in particular very low study retention rates. Analyses identified strong associations between incarceration and follow-up, possibly due to recidivism but also likely due to the structural challenges faced by Black men with a history of incarceration (Bowleg & Raj, 2012; Raj & Bowleg, 2012). Loss to follow-up included significant numbers of disconnected cellphones, highlighting the economic vulnerability of the population. Additional limitations include reliance on self-report, social desirability and recall biases, and limited generalizability to other groups of Black men such as sexual minorities, those who live in rural settings, or Black men of higher socioeconomic status (SES).

## Conclusion

This study advances empirical knowledge regarding the value of supporting the structural factors that affect the sexual health of Black heterosexual men. Despite null findings regarding HIV/STI incidence, significant improvements in employment and homelessness were observed. This study also suggests that STI clinics may offer a promising environment for targeting structural factors affecting health, such as housing and employment, through the provision of wraparound and social services for predominantly low SES Black heterosexual men in urban areas.

## Supplemental Material

MEN_Count_Outcompes_Paper_Web_Tables_1-4_3 – Supplemental material for Evaluation of the Making Employment Needs (MEN) Count Intervention to Reduce HIV/STI Risk for Black Heterosexual Men in Washington DC Click here for additional data file. Supplemental material, MEN_Count_Outcompes_Paper_Web_Tables_1-4_3 for Evaluation of the Making Employment Needs (MEN) Count Intervention to Reduce HIV/STI Risk for Black Heterosexual Men in Washington DC by Anita Raj, Nicole E. Johns, Florin Vaida, Lianne Urada, Jenne Massie, Jennifer B. Yore and Lisa Bowleg in American Journal of Men’s Health
