# Supplementary material for: Evaluation of the Making Employment Needs (MEN) Count Intervention to Reduce HIV/STI Risk for Black Heterosexual Men in Washington DC
Source: Am J Mens Health. 2019 Aug 21;13(4):1557988319869493. doi: 10.1177/1557988319869493 (PMC6709442; doi:10.1177/1557988319869493)
Supplement: MEN_Count_Outcompes_Paper_Web_Tables_1-4_3 – Supplemental material for Evaluation of the Making Employment Needs (MEN) Count Intervention to Reduce HIV/STI Risk for Black Heterosexual Men in Washington DC [file MEN_Count_Outcompes_Paper_Web_Tables_1-4_3.pdf]

Web Table 1. MEN Count outcomes at baseline, 6-month follow-up, and 12-month follow-up (N=454)[A]

	**Baseline**		**6-month follow-up**		**12-month follow-up**	
	Control	*Intervention*	*Control*	*Intervention*	*Control*	*Intervention*
	n (%)	*n (%)*	*n (%)*	*n (%)*	*n (%)*	*n (%)*
**Outcome**						
N	*227 (50.0)*	*227 (50.0)*	*105 (52.5)*	*95 (47.5%)*	*123 (50.6)*	*120 (49.4)*
Non-viral STI						
No	161 (75.9)	165 (75.0)	85 (86.7)	83 (92.2)	98 (86.7)	87 (83.7)
Yes	51 (24.1)	55 (25.0)	13 (13.3)	7 (7.8)	15 (13.3)	17 (16.3)
Sex Risk						
Very low	10 (5.6)	19 (10.2)	18 (21.7)	15 (19.2)	18 (17.8)	28 (30.8)
Low	36 (20.0)	48 (25.7)	29 (34.9)	32 (41.0)	40 (39.6)	26 (28.6)
Medium	104 (57.8)	87 (46.5)	28 (33.7)	25 (32.1)	34 (33.7)	30 (33.0)
High	30 (16.7)	33 (17.6)	8 (9.6)	6 (7.7)	9 (8.9)	7 (7.8)
Homeless in past 90 days						
No	122 (54.0)	111 (48.9)	70 (67.3)	67 (70.5)	91 (74.6)	84 (70.0)
Yes	104 (46.0)	116 (51.1)	32 (32.7)	28 (29.5)	31 (25.4)	36 (30.0)
Current employment						
Unemployed	149 (65.9)	158 (69.6)	65 (61.9)	48 (51.1)	62 (50.4)	59 (49.2)
Employed part time	43 (19.0)	50 (22.0)	20 (19.0)	23 (24.5)	23 (18.7)	32 (26.7)
Employed full time	34 (15.0)	19 (8.4)	20 (19.0)	23 (24.5)	38 (30.9)	29. (24.2)

[A]*Only non-missing values reported*

Web Table 2. MEN Count baseline demographics and outcomes by retention status (N=454)[A]

Characteristic	Lost to follow-up	Retained at 6- and/or 12-month follow-up	Chi2 p-value
	n (%)	n (%)	
N	*171 (37.7)*	*283 (62.3)*	
Demographics			
Age			0.86
18-24	52 (30.4)	82 (29.0)	
25-29	45 (26.3)	82 (29.0)	
30-39	48 (28.1)	72 (25.4)	
40-65	26 (15.2)	47 (16.6)	
Recruitment source			0.01
At clinic	105 (61.4)	138 (48.8)	
Friend	34 (19.9)	77 (27.2)	
Craigslist	16 (9.4)	48 (17.0)	
Flyer, CBO, other	16 (9.4)	20 (7.1)	
Education			0.15
Less than HS diploma/GED	26 (21.1)	38 (13.4)	
GED	31 (18.3)	47 (16.6)	
HS Diploma	51 (29.8)	93 (32.9)	
Some college or more	53 (31.0)	105 (37.1)	
Incarcerated ever			0.50
No	48 (28.1)	88 (31.1)	
Yes	123 (71.9)	195 (68.9)	
Incarcerated in past 90 days			0.01
No	136 (79.5)	250 (88.3)	
Yes	35 (20.5)	33 (11.7)	
Outcomes at baseline			
Non-viral STI			0.64
No	124 (74.3)	202 (76.2)	
Yes	43 (25.7)	63 (23.8)	
Sex Risk			0.74
Very low	11 (8.1)	18 (7.8)	
Low	30 (22.1)	54 (23.4)	
Medium	75 (55.1)	116 (50.2)	
High	20 (14.7)	43 (18.6)	
Homeless in past 90 days			0.39
No	83 (48.8)	150 (53.0)	
Yes	87 (51.2)	133 (47.0)	
Current employment			0.230.
Unemployed	122 (71.3)	185 (65.6)	
Employed part time	28 (16.4)	65 (23.1)	
Employed full time	21 (12.3)	32 (11.3)	

[A]*Only non-missing values reported*

Web Table 3. Dose effect analysis of MEN Count intervention on employment (reference is full-time employment, n=453) and homelessness (reference is no homelessness in prior 90 days, N=452).

	Employment[A]				Homelessness[B]	
	Unemployed		Employed Part-Time			
	AOR	95% CI	AOR	95% CI	AOR	95% CI
Dose-time interaction						
None	*Ref*	*Ref*	*Ref*	*Ref*	*Ref*	*Ref*
1 MEN Count session	0.71	0.28,1.81	0.82	0.30,2.23	0.99	0.38,2.60
2 MEN Count sessions	0.95	0.32,2.86	1.17	0.30,4.61	1.70	0.43,6.63
3 MEN Count sessions	0.37*	0.14,0.96	0.42	0.13,1.30	0.31*	0.10,0.96
Treatment						
Control	*Ref*	*Ref*	*Ref*	*Ref*	*Ref*	*Ref*
Intervention	1.48	0.81,2.73	1.88	0.93,3.81	1.24	0.66,2.35
Time						
Baseline	*Ref*	*Ref*	*Ref*	*Ref*	*Ref*	*Ref*
6-month follow-up	0.57*	0.36,0.91	0.67	0.36,1.26	0.27***	0.13,0.51
12-month follow-up	0.38***	0.24,0.60	0.53*	0.29,0.97	0.23***	0.12,0.44
Age						
18-24	*Ref*	*Ref*	*Ref*	*Ref*	*Ref*	*Ref*
25-29	0.72	0.41,1.29	0.75	0.41,1.37	1.92	0.91,4.08
30-39	1.24	0.66,2.36	0.70	0.35,1.38	2.84**	1.30,6.21
40-65	1.49	0.60,3.65	0.84	0.34,2.07	5.74***	2.20,14.99
Recruitment source						
At clinic	*Ref*	*Ref*	*Ref*	*Ref*	*Ref*	*Ref*
Friend	1.77	0.89,3.51	1.28	0.59,2.81	5.30***	2.51,11,18
Craigslist	0.70	0.64,1.34	1.36	0.70,2.64	1.90	0.81,4.44
Flyer, CBO, other	0.70	0.26,1.93	1.67	0.65,4.28	7.61**	2.33,24.85
Education						
Less than HS/GED	1.16	0.52,2.56	0.76	0.31,1.89	1.04	0.44,2.48
GED	0.54	0.26,1.12	0.60	0.26,1.38	2.43*	1.05,5.64
HS Diploma	*Ref*	*Ref*	*Ref*	*Ref*	*Ref*	*Ref*
Some college or more	0.58	0.32,1.03	0.91	0.51,1.62	0.63	0.31,1.29
Incarcerated in past 90 days at baseline						
No	*Ref*	*Ref*	*Ref*	*Ref*	*Ref*	*Ref*
Yes	1.66	0.73,3.78	1.23	0.53,2.84	1.49	0.68,3.28
Homeless in past 90 days at baseline						
No	*Ref*	*Ref*	*Ref*	*Ref*		
Yes	1.95**	1.18,3.24	1.17	0.69,2.00		
Employed at Baseline						
Unemployed					*Ref*	*Ref*
Employed Part-Time					0.28**	0.13,0.60
Employed Full Time					0.44	0.17,1.12

*p<0.05 **p<0.01 ***p<0.001

[A]Model clustered on individual.
[B]Model includes random effect on individual.

Web Table 4. MEN Count participant satisfaction survey results (N=277).[A]

	Control	Intervention	Total	Chi2 p-value control vs intervention
	n (%)	n (%)	n (%)	
N	*140*	*137*	*277*	
Did you participate in any MEN Count program sessions?				0.20
No	14 (10%)	8 (5.8%)	22 (7.9%)	
Yes	126 (90%)	129 (94.2%)	255 (92.1%)	
Did you participate in all three MEN Count program sessions?				0.76
No	65 (51.6%)	69 (53.5%)	134 (52.5%)	
Yes	61 (48.4%)	60 (46.5%)	121 (47.5%)	
Did the MEN Count program help you address concerns regarding your HIV risk and sexual health?				0.29
Very much	83 (66.4%)	86 (67.7%)	169 (67.1%)	
Somewhat	27 (21.6%)	34 (26.8%)	61 (24.2%)	
Not very much	7 (5.6%)	3 (2.4%)	10 (4%)	
Not at all	8 (6.4%)	4 (3.1%)	12 (4.8%)	
Did the MEN Count program help you address concerns regarding your risky substance use behaviors?				0.41
Very much	60 (62.5%)	69 (68.3%)	129 (65.5%)	
Somewhat	24 (25%)	24 (23.8%)	48 (24.4%)	
Not very much	4 (4.2%)	5 (5%)	9 (4.6%)	
Not at all	8 (8.3%)	3 (3%)	11 (5.6%)	
Did the MEN Count program help you address concerns regarding other health concerns?				0.81
Very much	69 (65.7%)	67 (59.8%)	136 (62.7%)	
Somewhat	21 (20%)	28 (25%)	49 (22.6%)	
Not very much	4 (3.8%)	5 (4.5%)	9 (4.1%)	
Not at all	11 (10.5%)	12 (10.7%)	23 (10.6%)	
Did the MEN Count program help you address concerns regarding your employment situation?				0.68
Very much	42 (37.5%)	44 (39.3%)	86 (38.4%)	
Somewhat	23 (20.5%)	24 (21.4%)	47 (21%)	
Not very much	9 (8%)	13 (11.6%)	22 (9.8%)	
Not at all	38 (33.9%)	31 (27.7%)	69 (30.8%)	
Did the MEN Count program help you address concerns regarding your housing situation?				0.45
Very much	32 (29.9%)	31 (29.2%)	63 (29.6%)	
Somewhat	21 (19.6%)	17 (16%)	38 (17.8%)	
Not very much	12 (11.2%)	20 (18.9%)	32 (15%)	
Not at all	42 (39.3%)	38 (35.8%)	80 (37.6%)	

	Control	Intervention	Total	**Chi2 p-value control vs intervention**
Did the MEN Count program help you maintain a healthier and happier relationship with your female sex partner(s)?				0.92
Very much	59 (47.2%)	54 (42.9%)	113 (45%)	
Somewhat	32 (25.6%)	34 (27%)	66 (26.3%)	
Not very much	11 (8.8%)	12 (9.5%)	23 (9.2%)	
Not at all	23 (18.4%)	26 (20.6%)	49 (19.5%)	
Did the MEN Count program help you reduce violence in your relationship(s) with female sex partners?				0.23
Very much	40 (69%)	28 (52.8%)	68 (61.3%)	
Somewhat	11 (19%)	11 (20.8%)	22 (19.8%)	
Not very much	2 (3.4%)	5 (9.4%)	7 (6.3%)	
Not at all	5 (8.6%)	9 (17%)	14 (12.6%)	
Did the MEN Count program help you build a healthier and happier relationship with your children?				0.38
Very much	45 (54.9%)	37 (52.9%)	82 (53.9%)	
Somewhat	19 (23.2%)	19 (27.1%)	38 (25%)	
Not very much	2 (2.4%)	5 (7.1%)	7 (4.6%)	
Not at all	16 (19.5%)	9 (12.9%)	25 (16.4%)	
How knowledgeable was the case manager about topics discussed in your MEN Count counseling sessions?				0.80
Very much	99 (79.2%)	106 (82.8%)	205 (81%)	
Somewhat	21 (16.8%)	16 (12.5%)	37 (14.6%)	
Not very much	2 (1.6%)	2 (1.6%)	4 (1.6%)	
Not at all	3 (2.4%)	4 (3.1%)	7 (2.8%)	
How useful were your action plans (created with your case manager) in helping you to achieve your program goals?				0.29
Very much	65 (51.6%)	75 (59.1%)	140 (55.3%)	
Somewhat	34 (27%)	36 (28.3%)	70 (27.7%)	
Not very much	10 (7.9%)	7 (5.5%)	17 (6.7%)	
Not at all	17 (13.5%)	9 (7.1%)	26 (10.3%)	
Did you feel that the case manager was someone like you, who could identify with you and could relate to you and your life experiences?				**0.005**
Very much	62 (50.8%)	92 (72.4%)	154 (61.8%)	
Somewhat	39 (32%)	25 (19.7%)	64 (25.7%)	
Not very much	10 (8.2%)	5 (3.9%)	15 (6%)	
Not at all	11 (9%)	5 (3.9%)	16 (6.4%)	
Did you feel comfortable with the case manager, able to share your feelings and concerns?				0.40
Very much	91 (74%)	103 (80.5%)	194 (77.3%)	
Somewhat	23 (18.7%)	21 (16.4%)	44 (17.5%)	
Not very much	6 (4.9%)	2 (1.6%)	8 (3.2%)	
Not at all	3 (2.4%)	2 (1.6%)	5 (2%)	

	Control	Intervention	Total	Chi2 p-value control vs intervention
Did you feel like the case manager understood you in terms of your needs related to the MEN Count program?				0.68
Very much	90 (73.2%)	102 (79.7%)	192 (76.5%)	
Somewhat	25 (20.3%)	20 (15.6%)	45 (17.9%)	
Not very much	5 (4.1%)	4 (3.1%)	9 (3.6%)	
Not at all	3 (2.4%)	2 (1.6%)	5 (2%)	
Overall, how would you rate the MEN Count program?				0.28
Excellent	76 (60.3%)	91 (68.9%)	167 (64.7%)	
Good	38 (30.2%)	35 (26.5%)	73 (28.3%)	
Fair	11 (8.7%)	6 (4.5%)	17 (6.6%)	
Poor	1 (0.8%)	0 (0%)	1 (0.4%)	
Would you recommend the MEN Count program to other men in your community?				0.70
Very much	118 (94.4%)	124 (93.2%)	242 (93.8%)	
Somewhat	7 (5.6%)	9 (6.8%)	16 (6.2%)	
Not very much	-	-	-	
Not at all	-	-	-	

[A]*Only non-missing values reported*
